# Medication-invariant resting aperiodic and periodic neural activity in Parkinson’s disease

**DOI:** 10.1111/psyp.14478

**Published:** 2023-11-08

**Authors:** Daniel J. McKeown, Manon Jones, Camilla Pihl, Anna J. Finley, Nicholas Kelley, Oliver Baumann, Victor R. Schinazi, Ahmed A. Moustafa, James F. Cavanagh, Douglas J. Angus

**Affiliations:** 1 Faculty of Society and Design, School of Psychology, Bond University, Gold Coast, Queensland, Australia; 2 Institute on Aging, University of Wisconsin-Madison, Madison, Wisconsin, USA; 3 School of Psychology, University of Southampton, Southampton, UK; 4 Department of Psychology, University of New Mexico, Albuquerque, New Mexico, USA

**Keywords:** aperiodic activity, cluster-based permutation tests, EEG, excitatory and inhibitory (E:I) balance, Parkinson’s disease

## Abstract

Parkinson’s disease (PD) has been associated with greater total power in canonical frequency bands (i.e., alpha, beta) of the resting electroencephalogram (EEG). However, PD has also been associated with a reduction in the proportion of total power across all frequency bands. This discrepancy may be explained by aperiodic activity (exponent and offset) present across all frequency bands. Here, we examined differences in the eyes-open (EO) and eyes-closed (EC) resting EEG of PD participants (*N* = 26) on and off medication, and age-matched healthy controls (CTL; *N* = 26). We extracted power from canonical frequency bands using traditional methods (total alpha and beta power) and extracted separate parameters for periodic (parameterized alpha and beta power) and aperiodic activity (exponent and offset). Cluster-based permutation tests over spatial and frequency dimensions indicated that total alpha and beta power, and aperiodic exponent and offset were greater in PD participants, independent of medication status. After removing the exponent and offset, greater alpha power in PD (vs. CTL) was only present in EO recordings and no reliable differences in beta power were observed. Differences between PD and CTL in the resting EEG are likely driven by aperiodic activity, suggestive of greater relative inhibitory neural activity and greater neuronal spiking. Our findings suggest that resting EEG activity in PD is characterized by medication-invariant differences in aperiodic activity which is independent of the increase in alpha power with EO. This highlights the importance of considering aperiodic activity contributions to the neural correlates of brain disorders.

## 1 | INTRODUCTION

More than ten million people worldwide are currently diagnosed with Parkinson’s disease (PD; Marras et al., 2018), experiencing significant motoric (e.g., tremors, impaired gait, rigidity, and bradykinesia) and non-motoric (e.g., depression, loss of smell, emotional dysregulation, and increased impulsivity) symptoms. Furthermore, the disease also affects carers, family members, and friends due to the significant physical and emotional burden associated with caring for the individual with PD (Blaszczyk, 2016; Marras et al., 2018).

Coinciding with the gradual loss of dopaminergic neurons in the substantia nigra that contribute to motoric symptoms, PD is also associated with atypical neural oscillations and atypical levels of excitatory and inhibitory neurotransmitters (Little & Brown, 2014; O’Gorman Tuura et al., 2018; Weinberger et al., 2006). These alterations in dopaminergic function are thought to cause widespread pathological neural oscillations across canonical frequency bands (e.g., delta; 1–4 Hz, theta; 4–7 Hz, alpha; 7–13 Hz, beta; 13–30 Hz, gamma; >30 Hz) during tasks (George et al., 2013; Singh et al., 2018) and in resting state recordings (Olde Dubbelink et al., 2013). Individuals with PD show elevated beta power compared to controls, with the most prominent differences typically observed over medio-central sites corresponding to the motor cortex. Although most studies have found that resting-state alpha is elevated in PD (Stoffers et al., 2007), others have reported a decrease (Caviness et al., 2016). Furthermore, abnormal oscillations (e.g., beta power and alpha/theta ratios) have been linked to several factors related to the severity of PD (Miladinović et al., 2021). These include disease stage, disease progression, and the severity of cognitive impairment (Jaramillo-Jimenez et al., 2021; Olde Dubbelink et al., 2013). In addition, pharmacological treatments alter oscillatory activity in PD. Specifically, cortical and subcortical alpha and beta power decrease following the administration of Levodopa (a dopamine precursor), and are associated with a reduction in symptom severity (Cao et al., 2020; Kuhn et al., 2006, 2009).

Recent studies have highlighted that these differences in canonical frequency power could be underpinned (fully or partially) by aperiodic (i.e., non-oscillatory) signal activity. Aperiodic activity, also called 1/*f* activity since its power decreases as a function of frequency, was historically not considered to be physiologically meaningful or likely to impact estimates of oscillatory activity (Donoghue, Haller, et al., 2020). Recent evidence suggests otherwise, and highlights its potential importance for interpreting encephalography (EEG) correlates of brain disorders, including PD.

First, aperiodic activity is physiologically meaningful. The aperiodic exponent (i.e., the slope of the power spectra) has been shown to reflect the balance of excitatory and inhibitory (E:I balance) neural activity in both cross-sectional (Brady & Bardouille, 2022), animal model (Gao et al., 2017), and human experimental studies (Bodizs et al., 2021; Waschke et al., 2021). Here, smaller (i.e., flatter) exponents are observed following manipulations that increase excitatory activity and/or decrease inhibitory activity, and larger (i.e., steeper) exponents have been observed following manipulations that increase inhibitory neural activity and/or decrease excitatory activity. Whereas, the size of the aperiodic offset (i.e., the height of the power spectra) is directly associated with the rate of neural spiking (Manning et al., 2009), and with blood-oxygen-dependent levels during task performance (Winawer et al., 2013). Second, aperiodic activity varies systematically and semi-independently of oscillations. The aperiodic exponent has been found to vary as a function of age (Finley et al., 2022; Hill et al., 2022; Voytek et al., 2015), clinical status (Karalunas et al., 2022; Shuffrey et al., 2022), cognitive function (Cross et al., 2022; Immink et al., 2021; Ostlund et al., 2021; Ouyang et al., 2020), and sleep state (Schneider et al., 2022). Third, these systematic differences in aperiodic activity could act as a substantial confound and create the appearance of systematic differences in oscillations in total power and ratio-based measures (Donoghue, Dominguez, et al., 2020; Donoghue, Haller, et al., 2020; Finley et al., 2022). As such, traditional quantification methods, including those that rely on the calculation of ratios, can result in a misestimation and misattribution of aperiodic activity and periodic activity.

Using EEG to determine the nature of aperiodic activity in PD offers unique insights into the pathophysiology of the disease and enhances the diagnostic potential of EEG. PD is characterized by the degeneration of dopaminergic neurons, resulting in increased firing of the subthalamic nucleus and imposed inhibition of thalamocortical drive, as well as reduced neural complexity (Gomez et al., 2011). Since this hypodopaminergic-state and reduction in neural complexity is characteristic of PD, this may be reflected in increases in the aperiodic exponent and offset. Indices of neural complexity and the capacity of neural systems to integrate and differentiate (such as the Lempel-Ziv algorithmic complexity index, LZC; Lempel & Ziv, 1976), are reduced in PD and may be associated with changes in aperiodic activity (Gomez et al., 2011; Rosenblum et al., 2023). Indeed, reductions in the LZC coincide with steeper exponents reflective of increased inhibition and a reduction in E:I balance, and reduced signal complexity (Rosenblum et al., 2023). Considering this, quantifying aperiodic activity in PD may be functionally significant and important for understanding the neuropathology of the disease.

Recent work has shown that PD is also associated with alterations in GABAergic neuron activity (Blaszczyk, 2016; O’Gorman Tuura et al., 2018) and that pharmaceutical treatment of these alterations (i.e., dopaminergic medications) may impact aperiodic activity by rebalancing E:I ratios (Belova et al., 2021; Wang et al., 2022). Convergent evidence from subdural electrophysiology (i.e., deep-brain stimulation, DBS; Belova et al., 2021; Darmani et al., 2023; Kim et al., 2022; Martin et al., 2018), neuroimaging, computational modeling (Moustafa et al., 2008), and drug trial (Song et al., 2021) studies, suggest that alterations in E:I balance contribute to impairments and alterations in the behavior of individuals with PD. Other studies have found that PD also alters the rate of neural spiking, particularly in the subthalamic nucleus (STN; Shimamoto et al., 2013), with the rate of spiking increasing with symptom severity and disease progression (Remple et al., 2011).

Considering this, the current study examined whether PD and sex- and age-matched healthy controls (CTL) differ in resting aperiodic activity, and the extent to which underlying aperiodic activity can account for differences in canonical alpha and beta frequency bands before (i.e., total alpha and beta) and following parameterization (i.e., parameterized alpha and beta). Using unpublished resting state data collected during a previous study (Cavanagh et al., 2018), we examine variation in the EEG spectra between PD on and off medication and CTL.

## 2 | METHOD

### 2.1 | Participants

Twenty-eight participants with PD were recruited from the Albuquerque, New Mexico community, and were paid $20 per hour for their participation. An equal number of age-and sex-matched CTL participants were also recruited (Cavanagh et al., 2018).^1^ The University of New Mexico Office of the Institutional Review Board approved the study and all participants provided informed consent. Two PD and CTL participants had insufficient clean EEG data and were excluded from analysis. EEG recordings were obtained from the PD participants on two occasions: once while on dopaminergic medication (ON), and once after a 15-hour overnight withdrawal from the dopaminergic medication (OFF). Demographic and diagnostic details for the retained participants (26 OFF/ON; 26 CTL) are presented in Table 1.

A battery of neuropsychological assessment tools was employed in both the PD and CTL cohorts. The Beck’s Depression Inventory (BDI) is a 21-item, self-reported inventory that measures symptoms of depression. Severity of depression symptoms are classified as minimal (0–9), mild (10–18), moderate (19–29), and severe (30–63). The Mini-Mental Status Exam (MMSE) is a cognitive screening tool that is used to assess cognitive impairment. A score of 30 is considered perfect, a score of 24 is recommended, and a score <24 indicates dementia. The North American Adult Reading Test (NAART) was used to characterize verbal intelligence. Each participant is presented with 50 words, which they need to pronounce correctly. One point is deducted from 50 for each word that is incorrectly pronounced. The Unified Parkinson’s Disease Rating Scale (UPDRS) was used to classify motor function disability (UPDRS III) when ON and OFF. The UPDRS III consists of 18 items with scores ranging from 0 to 132. Severity of motor disability is classified as mild (<32), moderate (33–58), and severe (>58; Sanchez-Ferro et al., 2018). That is, greater the score the greater the disability. UPDRS III motor scores were video recorded for each PD session and scored by a neurologist. There were no significant differences in demographic variables and psychological assessment scores between PD and CTL participants, nor severity of motor disability between ON and OFF (Table 1). Further detail on the medication status of the PD participants in the current study can be found in the Supplementary Material document.

### 2.2 | Experimental procedure

The procedures used in the current study are described in detail elsewhere (Cavanagh et al., 2018). PD participants visited the lab on two occasions, one-week apart. At one visit, PD participants were on medication, while on the other visit they were off medication. The order of ON and OFF visits was balanced across PD participants, with 13 PD participants on medication during their first visit and 13 PD participants off medication during their first visit. Recordings for both PD and CTL participants were collected at 9 AM. Neuropsychological assessments and questionnaires were completed by PD participants during their ON visit. In addition to the auditory oddball (Cavanagh et al., 2018), value of volition (Cavanagh et al., 2017), and cost of conflict (Singh et al., 2018) tasks completed by PD and CTL participants, one-minute of eyes-closed (EC) and eyes-open (EO) resting state EEG was recorded during each lab visit (Karalunas et al., 2022). These recordings taken prior to the experimental tasks. For these recordings, participants were asked to sit and rest quietly, first with EC, and then again with EO. All participants completed the EC and EO recordings in the same order.

### 2.3 | Physiological recording and processing

Continuous EEG was recorded using 64 active Ag/AgCl electrodes, arranged according to the international 10/20 system. EEG signals were sampled at 500 Hz and online band-pass filtered between 0.1 and 100 Hz using a Brain Vision amplifier. CPz and Afz were used as the reference and ground, respectively. Although vertical electrooculogram (VEOG) was recorded from auxiliary electrodes, these data were discarded during preprocessing. Preprocessing of EEG data was conducted using MATLAB (v2019a) and EEGLAB (v2021.0; Delorme & Makeig, 2004). Resting data were first referenced to the average of all scalp electrodes, and CPz was recreated. Data were then passed through the PREP pipeline, which we used to automatically identify and interpolate bad channels, reduce line-noise, and create a robust average reference (Bigdely-Shamlo et al., 2015). PREP processed data were high-pass (0.1 Hz) and notch filtered (60 Hz) to remove any remaining low-frequency and line-noise. We then used independent components analysis (ICA; Jung et al., 2000) and the Multiple Artifact Rejection Algorithm (MARA; Winkler et al., 2011; https://github.com/irenne/MARA) plugin to automatically identify and remove ocular, muscular, cardiovascular, and electrode artifacts. EC and EO data were then segmented into 2000 ms segments overlapping by 50%. Segments in which the voltage of any scalp channel deviated by ±150 μV were marked as artifactual and excluded from analysis. Two participants with <50% of non-artifactual EC or EO data during any lab visit were also excluded from analysis. Retained participants had an average of 97.05% (*SD* = 2.59%) non-artifactual data for EC recordings, and 97.43% (*SD* = 3.20%) for EO recordings. We then extracted the power spectra for EC and EO data from each channel for each participant using a Fast Fourier Transform (FTT) with a 2000 ms Hamming window, overlapping by 50%.

### 2.4 | Parameterization of power spectra

**FIGURE 1:**
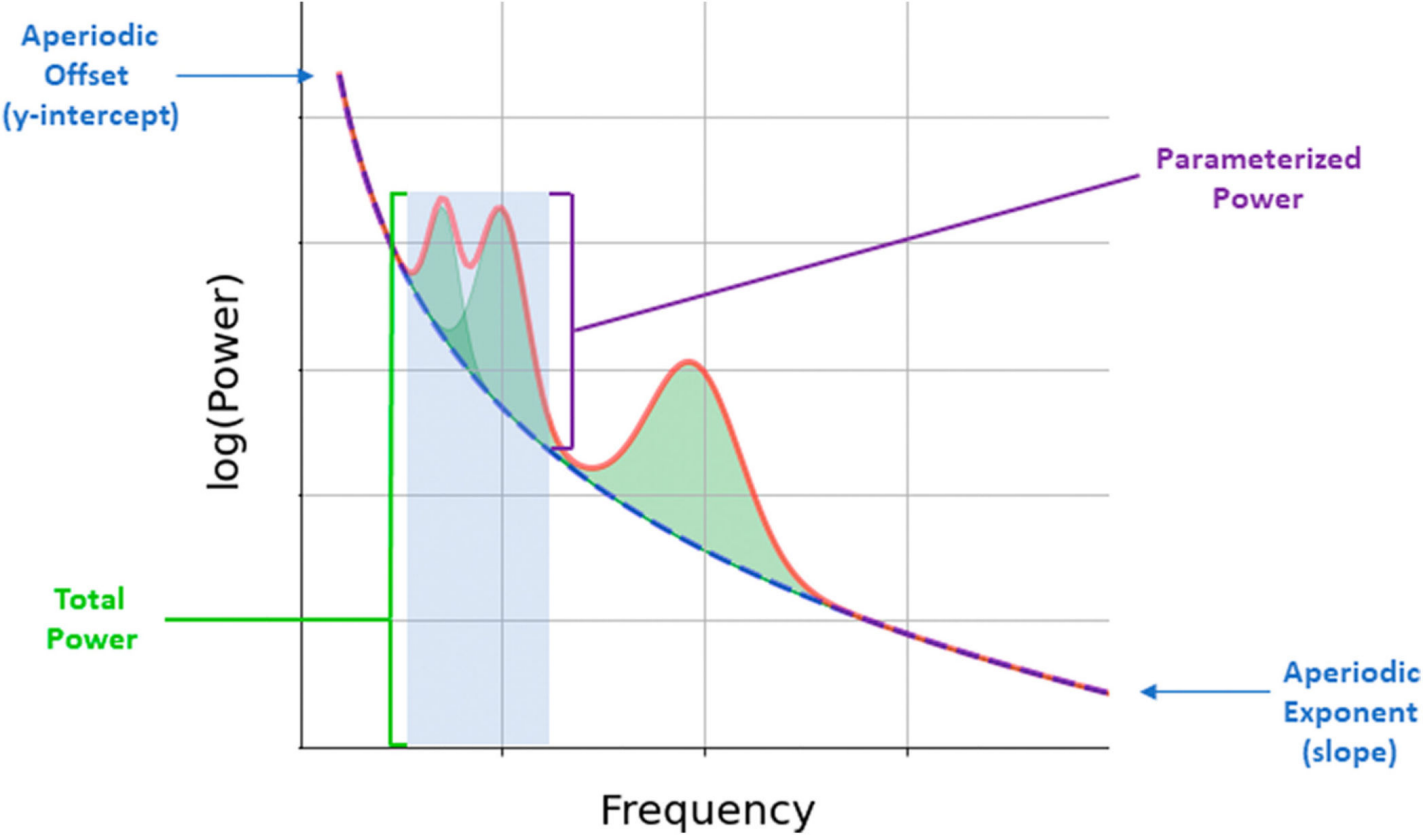
Example power spectral density (PSD) with strong alpha and beta peaks (green-shaded regions) over and above underlying aperiodic activity. Aperiodic offset represents the uniform shift of the PSD broadband, while the aperiodic exponent represents the 1/*f* nature of the PSD. Periodic power is traditionally characterized by the total power in the range of the canonical frequency band (blue-shaded region), but parameterized power represents the power of the periodic peak, beyond the aperiodic exponent.

We first extracted total alpha and total beta power using the traditional approach of taking the average power of the spectra between predefined canonical frequency bands (alpha: 8–13 Hz; beta: 13–30 Hz). Total power values were analyzed in linear space but are plotted in log-space to aid in visualization. To parameterize the power spectra into its aperiodic and periodic components (Figure 1), we used the *Specparam* algorithm (formally known as fitting oscillations and 1/*f* toolkit [FOOOF]; Donoghue, Haller, et al., 2020; https://github.com/fooof-tools/fooof). The *Specparam* algorithm uses an iterative process to quantify both non-oscillatory aperiodic activity—which follows a 1/*f*-like power distribution—and oscillatory activity that overlaps with the underlying 1/*f-*like power distribution. The algorithm initially fits an aperiodic component to a power spectrum, which models both the exponent (i.e., the slope) and the offset (i.e., the height) of the spectra. To identify oscillations, the algorithm then regresses out the initial aperiodic component from the power spectra, and iteratively detects peaks using Gaussian modeling in the now flattened spectra, stopping this process when a predetermined number of peaks has been reached. After combining and refitting the oscillatory peaks, the algorithm then refits the aperiodic component, combines both oscillatory and aperiodic fits, and estimates the goodness of fit between the original power spectra and the algorithm-derived model.

In the current study, and in keeping with previous research and recommendations (Finley et al., 2022; Ostlund et al., 2021), we parameterized the spectra from 2 to 40 Hz with a .25 Hz frequency resolution, using the following algorithm settings: peak width limits: 1–8; max number of peaks: 8; minimum peak height: 0.1; peak threshold: 2 *SD*; and aperiodic mode: fixed. For each channel by condition by participant combination, this approach yielded four parameters of interest: the aperiodic exponent, the aperiodic offset, parameterized alpha power, and parameterized beta power. Goodness-of-fit was evaluated using the mean *R*^2^ for each participant across EC and EO conditions. Fits were considered bad if they were >3*SD* below the mean fit across all channels, conditions, and participants. One CLT participant in the EO condition had a mean fit below the threshold of *R*^2^ = .939. As the inclusion or exclusion of this participant did not substantially alter any results, we opted to retain them in the analysis.^2^

### 2.5 | Statistical analysis

Differences between ON, OFF, and CTL participants in aperiodic and periodic activity, as well as EC and EO conditions within each group, were examined using non-parametric cluster-based permutation testing as implemented in MNE. This approach adjusts for Type 1 error rate increases that are intrinsic to making within-or between-subjects comparisons across multiple electrodes. All electrode sites were included in the cluster-based permutation analysis. Comparisons between ON and CTL and OFF and CTL were implemented using independent samples *t*-tests. Comparisons between ON and OFF, and between EC and EO, were implemented using a 1-sample *t*-test performed on subject and electrode level difference scores between ON and OFF conditions. For each of the cluster-based tests, we used a cluster threshold of *p* < .05 with 10,000 Monte Carlo permutations. Consistent with previous studies, we report the maximum *t* statistic within each cluster, the Cohen’s *d* averaged across all electrodes within each cluster, and the *p* value for the cluster (Merkin et al., 2023). If analyses did not yield any significant clusters, we reported the maximum *t* statistic and Cohens *d* averaged across all electrodes and omit any cluster *p* value.

## 3 | RESULTS

### 3.1 | Medication status and symptoms of motor disability

Motor symptom disability on and off medication was assessed in PD patients (Table 1). The severity of symptoms was not significantly different while on or off medication (ON: median = 20, 13–32.5; OFF: median = 22.5, 16–31.25; DIFF: median = −3, −7–3.25; *p* = .304). List of medication active ingredient and dosage can be found in Table 1 of Supplementary Material 2.

### 3.2 | Aperiodic activity

**FIGURE 2:**
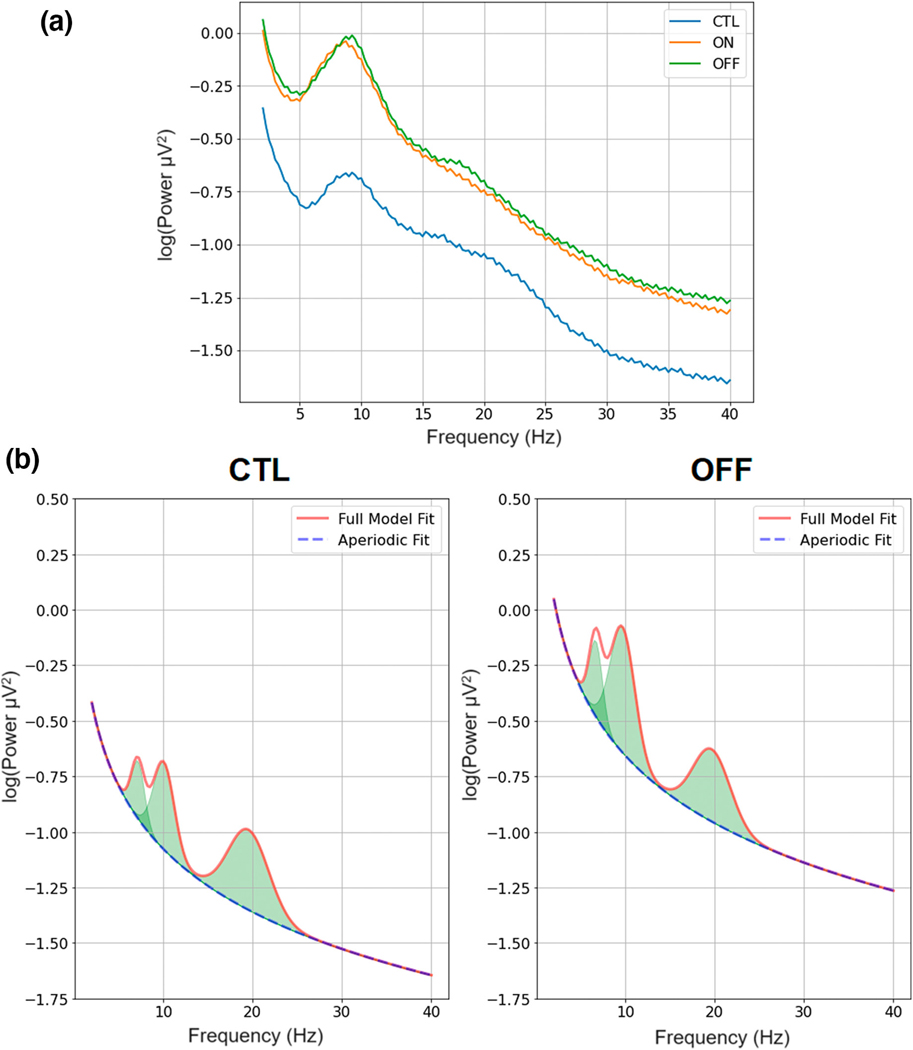
Average power spectral density (PSD) plots for healthy controls (CTL) and individuals with Parkinson’s disease while on (ON) and off medication (OFF) during eyes closed (EC) electroencephalography (EEG) recording (a). The parameterized PSD during EC clearly depicts the increased offset and steeper exponent in OFF compared to CTL (b).

**FIGURE 3:**
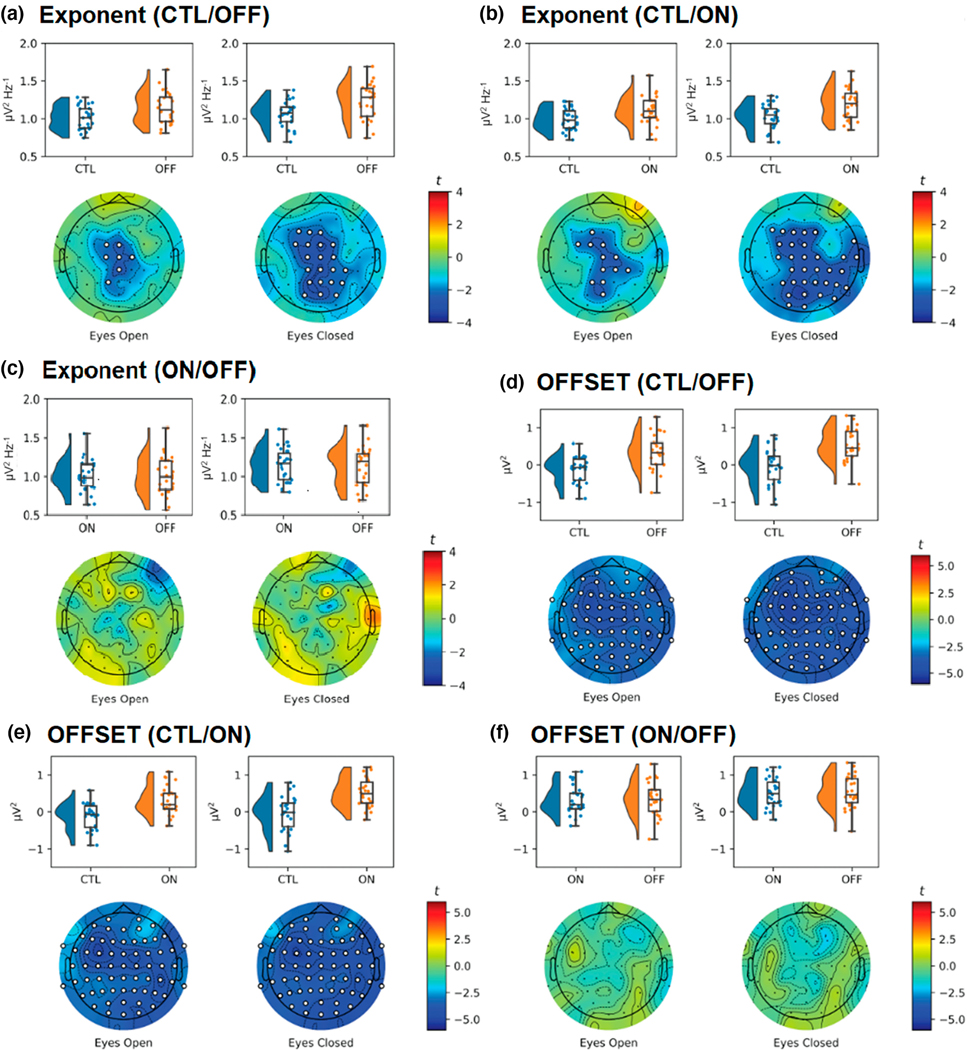
Raincloud and topographic plots comparing aperiodic activity in Parkinson’s disease (PD) and sex- and age-matched healthy controls (CTL). Raincloud plots include the smoothed distributions, boxplots, and individual data points included in each of the analyses. Topographic plots depict the scalp distribution of the t-statistic for the tests comparing CTL with off and on medication PD (OFF/ON), and ON with OFF. Electrodes with a *t*-statistic that exceeded the cutoff are masked in white. Panels a–c show differences in the aperiodic exponent for CTL versus OFF (a), CTL versus ON (b), and ON versus OFF (c), with greater (i.e., steeper) exponents in OFF/ON participants compared to CTL. Panels d–f show differences in the aperiodic offset for CTL versus OFF (d), CTL versus ON (e), and ON versus OFF (f) participants, with greater offsets for OFF/ON participants compared to CTL (*n* = 52).

Representative PSD plots are presented in Figure 2 that clearly depict the difference in EC aperiodic activity between CTL and PD. First, we explored differences in the aperiodic exponent (Figure 3a–c) and aperiodic offset (Figure 3d–f). Cluster-based tests indicated that PD tended to be associated with significantly larger (i.e., steeper) exponents. We observed significant differences between OFF and CTL in EC (*t*_max_ = 3.42, *d*_mean_ = 0.73, *p* = .024) but not in EO (*t*_max_ = 2.52, *d*_mean_ = 0.64, *p* = .060). Differences in the exponent between ON and CTL were observed in EO (*t*_max_ = 2.82, *d*_mean_ = 0.66, *p* = .033) and EC (*t*_max_ = 3.13, *d*_mean_ = 0.70, *p* = .014). There were no significant clusters when comparing ON with OFF in either EO (*t*_max_ = 1.93, *d*_mean_ = 0.00) or EC (*t*_max_ = 1.99, *d*_mean_ = 0.04). Differences in the exponent between CTL and ON and CTL and OFF participants were primarily observed over medial central locations, with differences only extending to parietal locations for EC metrics.

There were clear, scalp-wide differences between PD and CTL in the aperiodic offset, with larger (i.e., higher) offsets observed for OFF versus CTL (EO: *t*_max_ = 4.89, *d*_mean_ = 1.00, *p* < .001; EC: *t*_max_ = 5.01, *d*_mean_ = 1.12, *p* < .001), and ON versus CTL (EO: *t*_max_ = 5.11, *d*_mean_ = 1.00, *p* < .001; EC: *t*_max_ = 5.05, *d*_mean_ = 1.10, *p* < .001). As with the aperiodic exponent, there were no significant differences between ON and OFF (EO: *t*_max_ = 1.82, *d*_mean_ = 0.11; EC: *t*_max_ = 1.96, *d*_mean_ = 0.12).

### 3.3 | Alpha power

**FIGURE 4:**
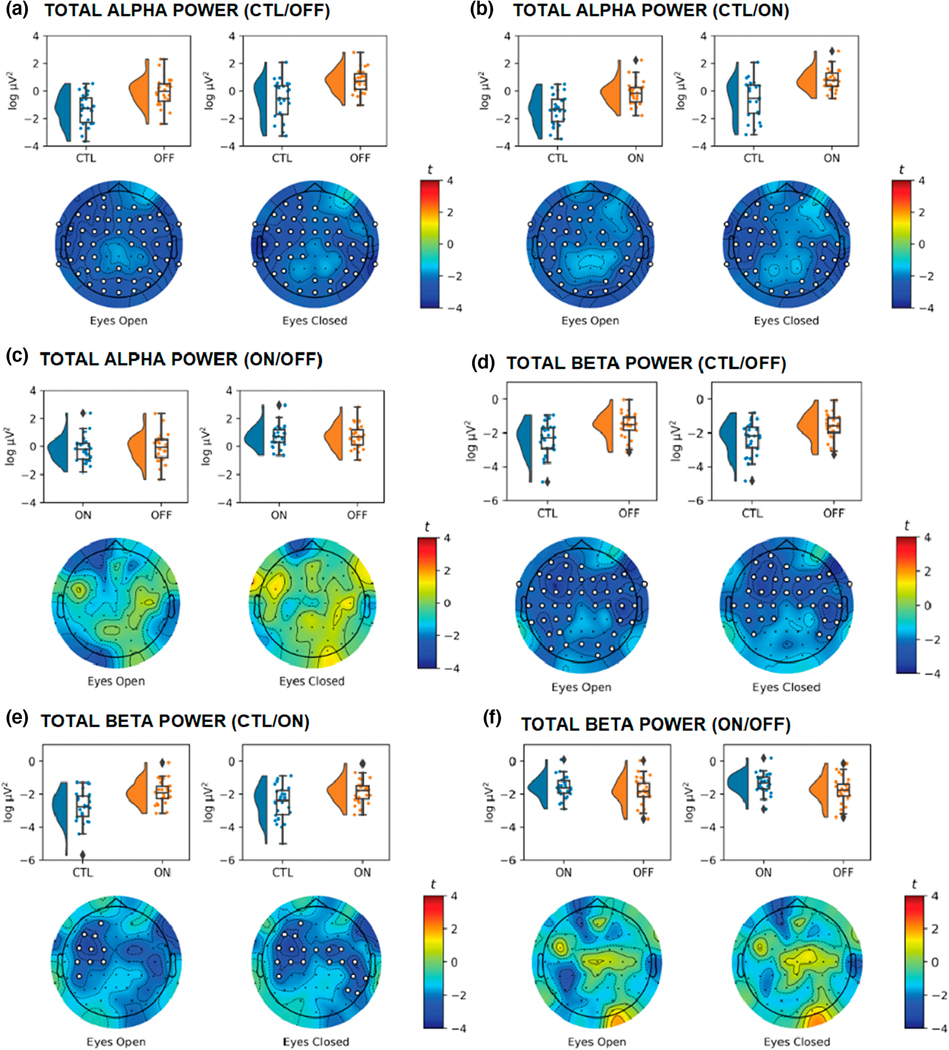
Raincloud and topographic plots comparing total alpha and total beta activity in Parkinson’s disease (PD) and sex- and age-matched healthy controls (CTL). Raincloud plots include the smoothed distributions, boxplots, and individual data points included in each of the analyses. Topographic plots depict the scalp distribution of the *t*-statistic for the tests comparing CTL with off and on medication PD (OFF/ON), and ON with OFF. Electrodes with a t-statistic that exceeded the cutoff are masked in white. Panels a–c show differences in total alpha power for CTL versus OFF (a), CTL versus ON (b), and ON versus OFF (c), with greater total alpha in OFF/ON participants compared to CTL. Panels d and e show differences in total beta power for CTL versus OFF (d), CTL versus ON (e), and ON versus OFF (f) participants, with greater total beta power for OFF/ON participants compared to CTL (*n* = 52).

**FIGURE 5:**
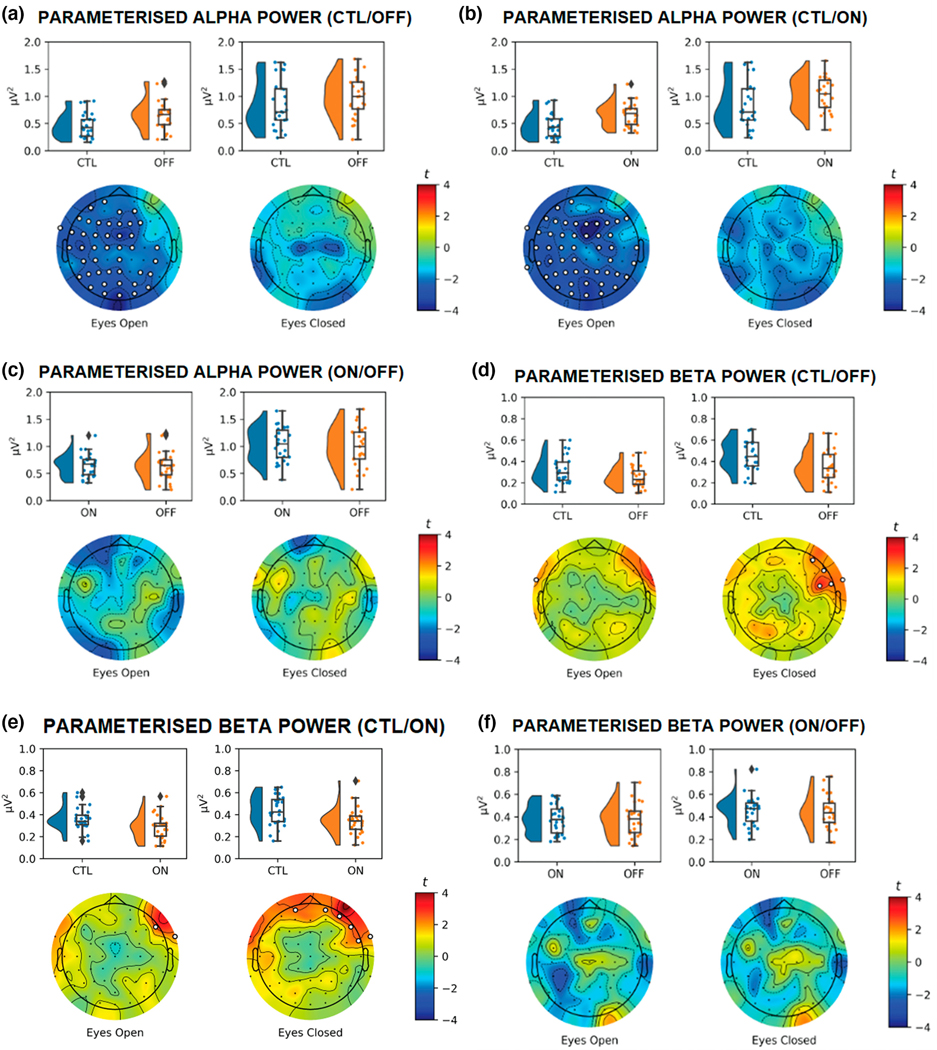
Raincloud and Topographic Plots Comparing Parameterized Alpha and Beta Activity in Parkinson’s disease (PD) and sex- and age-matched healthy controls (CTL). Raincloud plots include the smoothed distributions, boxplots, and individual data points included in each of the analyses. Topographic plots depict the scalp distribution of the *t*-statistic for the tests comparing CTL with OFF/ON, and ON with OFF. Electrodes with a *t*-statistic that exceeded the cutoff are masked in white. Panels a–c show differences in parameterized alpha power for CTL versus OFF (a), CTL versus ON (b), and ON versus OFF (c), with differences in power only observed between CTL and OFF/ON in eyes-open conditions. Panels d and e show differences in parameterized beta power for CTL versus OFF (d), CTL versus ON (e), and ON versus OFF (f) participants (*n* = 52).

Next, we explored the differences in total alpha power (Figure 4a–c) and parameterized alpha power (Figure 5a–c). There were widely distributed differences between PD and CTL for total alpha power, with greater total alpha power observed in PDs compared to CTL regardless of medication status or EO/EC condition (OFF vs. CTL EO: *t*_max_ = 3.39, *d*_mean_ = 0.73, *p* < .001; OFF vs. CTL EC: *t*_max_ = 3.86, *d*_mean_ = 0.72, *p* = .004; ON vs. CTL EO: *t*_max_ = 3.18, *d*_mean_ = 0.67, *p* = .001; OFF vs. CTL EC: *t*_max_ = 3.61, *d*_mean_ = 0.71, *p* = .005). There were no significant differences between ON and OFF in EO (*t*_max_ = 2.62, *d*_mean_ = 0.50, *p* = .129) or EC (*t*_max_ = 1.89, *d*_mean_ = 0.01) conditions.

Although parameterized alpha power was greater for OFF and ON compared to CLTs, these differences were only significant in the EO condition (OFF vs. CTL: *t*_max_ = 3.79, *d*_mean_ = 0.72, *p* = .012; ON vs. CTL: *t*_max_ = 4.37, *d*_mean_ = 0.77, *p* = .006). There were no significant differences between OFF and CTL (*t*_max_ = 2.59, *d*_mean_ = 0.73, *p* = .287) and ON and CTL (*t*_max_ = 2.27, *d*_mean_ = 0.64, *p* = .134) in the EC condition, or between ON and OFF in EO (*t*_max_ = 1.55, *d*_mean_ = 0.00) or EC (*t*_max_ = 2.37, *d*_mean_ = 0.47, *p* = .695).

### 3.4 | Beta power

We examined differences in total (Figure 4d–f) and parametrized (Figure 5d–f) beta power. Total beta power was consistently greater for PD participants versus CTL. These differences were observed when comparing OFF with CTL (EO: *t*_max_ = 3.47, *d*_mean_ = 0.72, *p* = .005; EC: *t*_max_ = 3.37, *d*_mean_ = 0.73, *p* = .009), and when comparing ON with CTL (EO: *t*_max_ = 3.34, *d*_mean_ = 0.73, *p* = .031; EC: *t*_max_ = 3.39, *d*_mean_ = 0.71, *p* = .021). There were no differences in total beta as a function of medication status (EO: *t*_max_ = 2.55, *d*_mean_ = 0.47, *p* = .173; EC: *t*_max_ = 2.54, *d*_mean_ = 0.49, *p* = .347).

The cluster-based differences observed for total beta were not present when examining parametrized beta. Although there was greater parameterized beta for CTL versus PD participants over right fronto-polar electrodes in the EC conditions (Figure 5d,e), these clusters did not reach significance. That is, after removing the aperiodic component, there were no significant differences in beta power for CTL versus OFF in EO (*t*_max_ = 2.05, *d*_mean_ = 0.58, *p* = .453) or EC (*t*_max_ = 2.91, *d*_mean_ = 0.67, *p* = .077), CTL versus ON in the EO (*t*_max_ = 2.42, *d*_mean_ = −0.64, *p* = .203) or EC (*t*_max_ = 2.92, *d*_mean_ = −0.64, *p* = .070). As with other measures, we did not observe a difference in parametrized beta or for ON versus OFF in the EO (*t*_max_ = 1.41, *d*_mean_ = 0.04) or EC (*t*_max_ = 1.82, *d*_mean_ = 0.08) conditions.

### 3.5 | Differential EEG parameters between EC and EO conditions

**FIGURE 6:**
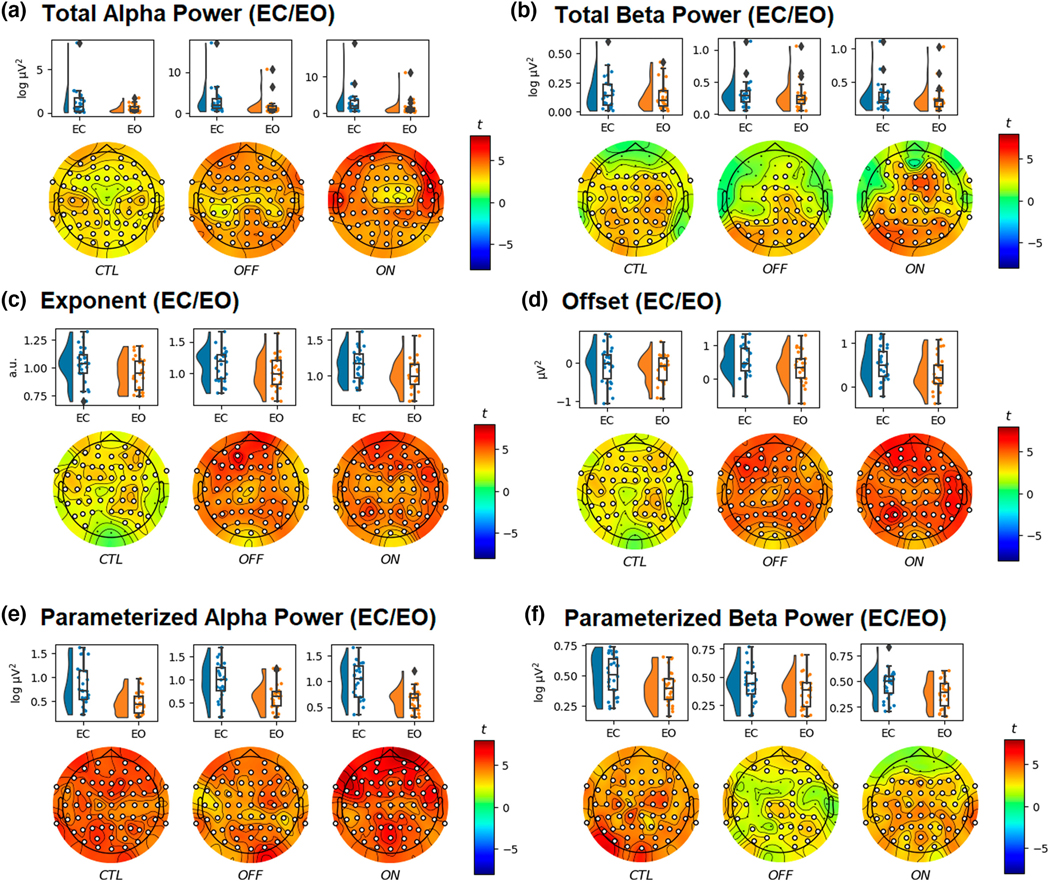
Raincloud and topographic plots comparing total alpha power (a), total beta power (b), aperiodic exponent (c), aperiodic offset (d), parameterized alpha power (e), and parameterized beta power (f), in age-matched healthy controls (CTL), and Parkinson’s disease off (OFF) and on medication (ON), during eyes closed (EC) and eyes open (EO). Raincloud plots include the smoothed distributions, boxplots, and individual data points included in each of the analyses. Topographic plots depict the scalp distribution of the *t*-statistic. Electrodes with a t-statistic that exceeded the cutoff are masked in white (*n* = 52).

Lastly, we examined differences in the periodic and aperiodic measures between the EC and EO conditions within CTL, OFF, and ON groups. Total alpha power (Figure 6a) was greater during EC for CTL (*t*_max_ = 3.83, *d*_mean_ = 0.54, *p* < .001), OFF (*t*_max_ = 5.05, *d*_mean_ = 0.74, *p* = .001), and ON groups (*t*_max_ = 6.75, *d*_mean_ = 0.85, *p* < .001). This was also seen for total beta power (Figure 6b) in the CTL (*t*_max_ = 4.26, *d*_mean_ = 0.61, *p* = .003), OFF (*t*_max_ = 3.99, *d*_mean_ = 0.60, *p* = .019), and ON groups (*t*_max_ = 5.12, *d*_mean_ = 0.74, *p* < .001). The aperiodic exponent (Figure 6c) was greater during the EC condition in CTL (*t*_max_ = 3.52, *d*_mean_ = 0.53, *p* = .01), OFF (*t*_max_ = 6.70, *d*_mean_ = 0.89, *p* = .002), and ON groups (*t*_max_ = 5.85, *d*_mean_ = 0.91, *p* = .003). Similarly, the aperiodic offset (Figure 6d) was greater during the EC condition in CTL (*t*_max_ = 3.84, *d*_mean_ = 0.53, *p* = .009), OFF (*t*_max_ = 6.04, *d*_mean_ = 0.92, *p* = .003), and ON groups (*t*_max_ = 6.61, *d*_mean_ = 1.03, *p* = .004). Following parameterization, parameterized alpha power (Figure 6e) was greater during the EC condition in CTL (*t*_max_ = 5.94, *d*_mean_ = 1.00, *p* < .001), OFF (*t*_max_ = 5.84, *d*_mean_ = 0.85, *p* < .001), and ON groups (*t*_max_ = 7.16, *d*_mean_ = 1.08, *p* < .001). This was also seen for parameterized beta power (Figure 6f) in the CTL (*t*_max_ = 6.05, *d*_mean_ = 0.83, *p* < .001), OFF (*t*_max_ = 3.51, *d*_mean_ = 0.53, *p* = .001), and ON groups (*t*_max_ = 5.09, *d*_mean_ = 0.69, *p* < .001).

## 4 | DISCUSSION

In this study, we examined differences in alpha and beta band power before isolation from aperiodic activity (unparameterized) and when accounting for aperiodic activity (parameterized) in the resting state EEG of PD patients compared to CTL. We also explored the effect of medication status of PD patients on these EEG parameters. We found that individuals with PD showed higher exponents and larger offsets compared to CTL during both EO and EC conditions, regardless of medication status. Additionally, PD patients exhibited higher total alpha and beta power during both eye conditions, but these differences were reduced when accounting for aperiodic activity during both eye conditions for beta power, and only during EC for alpha power. These findings indicate that changes in canonical neural oscillatory activity in individuals with PD are primarily driven by changes in E:I balance favoring greater inhibition (aperiodic exponent) and greater neural spiking rate (aperiodic offset) when at rest.

### 4.1 | Aperiodic activity in Parkinson’s disease

Converging evidence indicates that impairment in neurotransmitter (dopamine and GABA) balance contributes to alterations in the behavior of individuals with PD (Song et al., 2021). Indeed, impairment in dopaminergic and GABAergic neuronal activity are prominent in PD (Blaszczyk, 2016; Meder et al., 2019; O’Gorman Tuura et al., 2018). A reduction in dopamine concentrations in the substantia nigra pars compacta and subthalamic nucleus (STN), key components of the basal ganglia and integral for motor control, has been found to result in over-inhibition and slowing of movement (i.e., bradykinesia; Chu et al., 2017; Meder et al., 2019). Similarly, reductions in GABA concentrations in the basal ganglia, motor cortex, and occipital cortex are associated with motor (resting tremor, reemergent tremor, and bradykinesia; van Nuland et al., 2020) and non-motor (hallucinations; Firbank et al., 2018) symptoms, the severity of which is determined by the progression of PD. PD also alters the rate of neural spiking, particularly in the STN (Shimamoto et al., 2013), with the rate of spiking increasing with symptom severity and disease progression (Remple et al., 2011). Concerning GABAergic neuronal activity, these findings are not surprising given the major GABAergic pathways in the basal ganglia, in contrast to other subcortical structures (Caligiore et al., 2016). Despite discrepancies in GABA changes in PD being prominent, they are likely related to differences in disease status, the subtype of PD group under investigation (i.e., tremor-dominant vs akinesia-dominant), and PD symptoms under investigation (axial vs. cardinal), as well as medication status.

The quantification of non-oscillatory aperiodic activity provides insight into local and widespread excitatory and inhibitory neural activity (Finley et al., 2022). Furthermore, the ability to infer E:I balance (aperiodic exponent), rate of neural spiking (aperiodic offset), and underlying changes in neurotransmitter availability, is particularly important for understanding the neuropathology of PD (Gao et al., 2017). Of the few studies that have characterized changes in aperiodic activity in PD, investigation have primarily been localized to deep brain regions using DBS (i.e., STN; Belova et al., 2021;Darmani et al., 2023; Kim et al., 2022; Martin et al., 2018). In these studies, the aperiodic exponent positively correlated with DBS treatment at rest (greater exponent slope from 2 to 6 months of DBS; Darmani et al., 2023), negatively correlated with voluntary muscle contractions of the finger flexors and tibialis anterior muscle independent of medication status (reduced exponent slope indicating greater excitation; Belova et al., 2021), and correlated with dopamine depletion (Kim et al., 2022). Together, these findings highlight the physiological relevance of aperiodic activity in the activation of the STN in PD. Consequently, increased inhibition of the STN (indicated by a greater exponent slope) has been identified as a potential mechanism for the efficacy of long-term DBS in the treatment of PD and as a biomarker for disease progression (Darmani et al., 2023; Kim et al., 2022; Martin et al., 2018).

Although changes in aperiodic activity during DBS provide evidence for the STN being a potential neural generator for aperiodic activity, its pertinence is limited by the surgical invasiveness of the procedure. Of the few studies assessing aperiodic activity noninvasively with scalp EEG in PD (Rosenblum et al., 2023; Wang et al., 2022), Wang et al. (2022) determined differences in the aperiodic exponent and offset in 15 PD on and off medication, compared to 16 CTL. Although statistically underpowered, dopaminergic medication increased the exponent and offset in patients with PD compared to off medication, but they were not different from CTL. These differences were greatest in the bilateral central brain regions (C4, C3, CP5, and FC5 regions of the scalp EEG) and was concluded to be due to the rebalancing effect of dopaminergic medication on E:I balance in the basal ganglia. Similarly, Rosenblum et al. (2023) demonstrated that PD patients show steeper aperiodic exponents compared to healthy controls which serves as a marker of neural network dysfunction (reduced E:I ratio), specifically an indication of the hypodopaminergic-state of the population.

In the current study, a greater aperiodic offset was observed in PD individuals compared to CTL during EO and EC conditions, while the aperiodic exponent was only significant during the EC condition. Furthermore, these findings were sufficiently statistically powered and observed in a larger PD population than previously investigated (Rosenblum et al., 2023; Wang et al., 2022), independent of medication status (Darmani et al., 2023), found throughout multiple brain regions, including the bilateral central regions previously documented (Wang et al., 2022), and aligned with those documented during DBS (Belova et al., 2021; Darmani et al., 2023). That is, increases in the aperiodic offset and exponent may reflect the hypodopaminergic state characteristic of PD. This provides evidence of the potential efficacy of using non-invasive scalp EEG as an alternative to invasive subdural electrodes and sensing-enabled implantable pulse generators when identifying disease and treatment biomarkers of PD.

### 4.2 | Periodic alpha and beta activity in Parkinson’s disease

Periodic activity has traditionally been assessed by the quantification of neural oscillatory activity in canonical frequency bands (i.e., alpha and beta band activity) while not controlling for aperiodic activity. This is problematic, as underlying aperiodic activity may result in misinterpretation of periodic activity. Considering this, previous conclusions on the changes in alpha and beta activity in PD may in fact be due to changes in the exponent and/or offset of aperiodic activity. This likely contributes to the conflicting findings of periodic activity assessment in PD. Indeed, increases in the power and synchronization of oscillations in the beta band are frequently reported (Canessa et al., 2016; Little & Brown, 2014; O’Gorman Tuura et al., 2018; Weinberger et al., 2006) but have also been reported to decrease in line with disease severity (Vinding et al., 2020). Whereas, alpha band power is low in bilateral parietooccipital locations in PD individuals with dementia (Yilmaz et al., 2020) and higher without dementia (Stoffers et al., 2007). This highlights the necessity for parameterization of these alpha and beta bands to accurately reflect changes in oscillatory activity in PD.

Once alpha and beta activity are parameterized, mixed responses are documented in PD. Although total power and parameterized power have been reported to be comparable in both alpha and beta bands (Belova et al., 2021; Wang et al., 2022), parameterized beta power has been reported to be more sensitive to changes in local field potential features (Belova et al., 2021) and is significantly more accurate at estimating the beta biomarker of PD symptom severity (Martin et al., 2018). Indeed, the correlation between aperiodic activity and motor symptoms of PD (bradykinesia and rigidity) is most evident once beta band power is isolated from the aperiodic exponent and offset (Martin et al., 2018). In the current study, total alpha and beta power were significantly greater in PD during both conditions and independent of medication status. Once isolated from aperiodic activity, parameterized alpha and beta power ceased to show significance. This indicates that the change in oscillatory activity between PD and CTL can be accounted for by a change in underlying aperiodic activity and not alpha and beta power. That is, individuals with PD exhibited a greater proportion of inhibitory neural balance and an increase in the rate of neural spiking in the current study, mechanisms of which are independent of periodic activity (Gao et al., 2017).

### 4.3 | Dopaminergic medication and EEG activity

In the current study, no differences in periodic nor aperiodic activity were observed in PD patients between on or off medication. Furthermore, severity of motor disability symptoms did not between on and off medication. This suggests that dopaminergic medication did not change EEG signatures in our PD cohort. Conversely, Wang et al. (2022) found that dopaminergic medication increased the aperiodic exponent and offset, specifically in the bilateral central brain regions, and that this aperiodic activity in ON did not differ with CTL. The increase in aperiodic activity was suggested to be due to the recruitment of inhibitory inputs from the globus pallidus external to the STN. To date, six main types of medications are available to alleviate symptoms of PD, including levodopa, dopamine agonists, inhibitors of enzymes that inactive dopamine (i.e., monoamine oxidase type B inhibitors [MAOB] and catechol-O-methyl transferase inhibitors [COMT]), anticholinergics, and amantadine (Connolly & Lang, 2014). Considering our PD population were also on a variety of medication (see Supplementary Material document), it is likely the variability in symptomatology whilst off medication contributed to the similarities in periodic and aperiodic activity between PD and healthy CTL in the current study.

### 4.4 | Implications and limitations

Although our findings are consistent with those of DBS (Belova et al., 2021; Darmani et al., 2023; Kim et al., 2022; Martin et al., 2018), oscillatory measurements derived from EEG are hard to localize. Although our study reveals atypical exponents in PD, we cannot say which structures acted as neural generators for aperiodic activity in our cohort. Nonetheless, the use of EEG provides a non-invasive approach to characterize aperiodic and periodic activity biomarkers in PD. This study provides evidence that the contribution of aperiodic activity to canonical alpha and beta band power needs to be considered carefully, and previous models using MEG/EEG need to be reevaluated. Indeed, differences in alpha and beta band power may be artifactual and instead driven by differences in the aperiodic exponent and offset.

*Specparam* is currently one of the most used parameterizations tool to separate periodic and aperiodic activity (Brady & Bardouille, 2022; Finley et al., 2022; Hill et al., 2022; Merkin et al., 2023). However, there are some inherent methodological considerations that need to be addressed. The EEG spectra is made up of multiple sources of noise, those of which are not captured by the parameterization of periodic or aperiodic activity. A component of this Gaussian noise is termed white noise which appears as a high-frequency horizontal line (slope *β* = 0) in the double-log space, which can disrupt the 1/*f* power law and may impact the estimation of the aperiodic exponent. This can result in overestimation and the presence of negative power values (Barry & De Blasio, 2021; Gerster et al., 2022). Although recent research using techniques that disentangle these estimates have produced similar results as ours (Rosenblum et al., 2023), future research needs to consider the influence of white noise when estimating 1/*f* activity. In the current study, great lengths were taken to ensure that the model fits of the PSD were excellent. This was achieved by following current recommendations for data cleaning prior to parameterization of the PSD (i.e., ICA and MARA analyses; Gerster et al., 2022). Furthermore, no negative power values were observed following parameterization. Because of this, we determine that the measures of aperiodic activity in the current study are reliable.

Interestingly, we examined that periodic and aperiodic activity differed between the groups when EC and EO, especially in the PD group. That is, periodic and aperiodic activity were greater with EC than EO. Since alpha rhythm is dependent on whether an individual’s eyes are open and closed, and since alpha rhythm also impacts aperiodic activity (Hohaia et al., 2022), future work needs to uncover what impact eye status has on aperiodic activity. Although we recommend acquiring EEG activity in PD during EC, the within-subject contrast between EC and EO may help isolate this effect. This may also benefit the comparison of EEG with other modalities of assessing brain activity (i.e., resting functional magnetic resonance imaging) that are typically employed in PD.

## 5 | CONCLUSION

When parametrizing alpha and beta band power derived from EEG at rest in individuals with PD and CTL, the increase in total alpha and beta power is in fact due to an increase in the aperiodic exponent (greater inhibition) and an increase in the aperiodic offset (greater neural spiking rate) and not in fact due to changes in parameterized alpha or beta band power. Furthermore, changes in aperiodic activity were independent of medication status. These findings correlate with those of previous DBS findings, and in doing so, provide a non-invasive approach to assess underlying changes in E:I balance and PD biomarkers.

## Supplementary Material

Supplementary Materials **Figure S1**. Correlation matrix for PD patients during EC condition. Reported are the *r* values.
**Figure S2**. Correlation matrix for PD patients during EO condition. Reported are the *r* values.
**Figure S3**. Correlation matrix for CTL during EC condition (top panel) and EO condition (bottom panel). Reported are the *r* values.
**Table S1**. Medication status of Parkinson’s disease participants.

## Figures and Tables

**TABLE 1:** Descriptive statistics for PD and CTL participants.

	PD (*n* = 26)	CTL (*n* = 26)	*t*	*p*
Age	71.5 (68 to 76)	69 (64–75.5)	0.20	.841
Sex = male (%)	15 (57.7)	16 (61.5)	0.28	.779
Years of education	18 (16 to 20)	16 (16 to 19)	0.88	.381
BDI	5.5 (3.25 to 10.25)	4.5 (.25 to 7)	1.56	.125
MMSE	29 (28 to 30)	29 (28 to 30)	0.55	.585
NAART	46.5 (42 to 54)	48 (44.75 to 51.25)	0.32	.749
Total daily LED mg	600 (384.5 to 775)	–	–	–
Years since diagnosis	4 (2.75 to 7.5)	–	–	–
UPDRS III ON	20 (13 to 32.5)	–	–	–
UPDRS III OFF	22.5 (16 to 31.25)	–	–	–
UPDRS III DIFF	−3 (−7 to 3.25)	–	1.05	.304

*Note*: Data are presented as medians and (Q1–Q3), except for sex which is presented as *n* of males (%).

Abbreviations: BDI, Beck Depression Inventory; CTL, age-matched controls; LED, L-Dopa equivalence dose in mg; MMSE, mini-mental state exam; NAART, North American adult reading test; ON/OFF, when on or off medication; PD, Parkinson’s disease; UPDRS III, United Parkinson’s disease rating scale (motor).

## Data Availability

The raw data used in this study are available at http://predict.cs.unm.edu/downloads.php, accession # d002. Processed data and for all analyses and plots are publicly available at https://github.com/MindSpaceLab/Parkinsons_Aperiodic_Periodic.

## Footnotes

1 Raw data for PD and CTL participants are available at http://predict.cs.unm.edu/downloads.php, accession # d002.

2 Exclusion of this participant only changed one result: Once excluded, parametrized beta power in the EC condition over a fronto-polar cluster was significantly greater in CTL participants compared to ON participants (*t*_max_ = 3.13, *d*_mean_ = 0.71, *p* = .041). No other results were altered.

## References

[R1] Barry RJ, & De Blasio FM (2021). Characterizing pink and white noise in the human electroencephalogram. Journal of Neural Engineering, 18(3), 034001. 10.1088/1741-2552/abe399 33545698

[R2] Belova EM, Semenova U, Gamaleya AA, Tomskiy AA, & Sedov A. (2021). Voluntary movements cause beta oscillations increase and broadband slope decrease in the subthalamic nucleus of parkinsonian patients. European Journal of Neuroscience, 53(7), 2205–2213. 10.1111/ejn.14715 32141151

[R3] Bigdely-Shamlo N, Mullen T, Kothe C, Su KM, & Robbins KA (2015). The PREP pipeline: Standardized preprocessing for large-scale EEG analysis. Frontiers in Neuroinformatics, 9, 16. 10.3389/fninf.2015.00016 26150785 PMC4471356

[R4] Blaszczyk JW (2016). Parkinson’s disease and neurodegeneration: GABA-collapse hypothesis. Frontiers in Neuroscience, 10, 269. 10.3389/fnins.2016.00269 27375426 PMC4899466

[R5] Bodizs R, Szalardy O, Horvath C, Ujma PP, Gombos F, Simor P, Pótári A, Zeising M, Steiger A, & Dresler M. (2021). A set of composite, non-redundant EEG measures of NREM sleep based on the power law scaling of the Fourier spectrum. Scientific Reports, 11(1), 2041. 10.1038/s41598-021-81230-7 33479280 PMC7820008

[R6] Brady B, & Bardouille T. (2022). Periodic/aperiodic parameterization of transient oscillations (PAPTO)-implications for healthy ageing. NeuroImage, 251, 118974. 10.1016/j.neuroimage.2022.118974 35131434

[R7] Caligiore D, Helmich RC, Hallett M, Moustafa AA, Timmermann L, Toni I, & Baldassarre G. (2016). Parkinson’s disease as a system-level disorder. NPJ Parkinson’s Disease, 2(1), 16025. 10.1038/npjparkd.2016.25 PMC5516580 28725705

[R8] Canessa A, Pozzi NG, Arnulfo G, Brumberg J, Reich MM, Pezzoli G, Ghilardi MF, Matthies C, Steigerwald F, Volkmann J, & Isaias IU (2016). Striatal dopaminergic innervation regulates subthalamic beta-oscillations and cortical-subcortical coupling during movements: Preliminary evidence in subjects with Parkinson’s disease. Frontiers in Human Neuroscience, 10, 611. 10.3389/fnhum.2016.00611 27999534 PMC5138226

[R9] Cao C, Li D, Zhan S, Zhang C, Sun B, & Litvak V. (2020). L-dopa treatment increases oscillatory power in the motor cortex of Parkinson’s disease patients. Neuroimage: Clinical, 26, 102255. 10.1016/j.nicl.2020.102255 32361482 PMC7195547

[R10] Cavanagh JF, Kumar P, Mueller AA, Richardson SP, & Mueen A. (2018). Diminished EEG habituation to novel events effectively classifies Parkinson’s patients. Clinical Neurophysiology, 129(2), 409–418. 10.1016/j.clinph.2017.11.023 29294412 PMC5999543

[R11] Cavanagh JF, Mueller AA, Brown DR, Janowich JR, Story-Remer JH, Wegele A, & Richardson SP (2017). Cognitive states influence dopamine-driven aberrant learning in Parkinson’s disease. Cortex, 90, 115–124. 10.1016/j.cortex.2017.02.021 28384481 PMC5470538

[R12] Caviness JN, Utianski RL, Hentz JG, Beach TG, Dugger BN, Shill HA, Driver-Dunckley ED, Sabbagh MN, Mehta S, & Adler CH (2016). Differential spectral quantitative electroencephalography patterns between control and Parkinson’s disease cohorts. European Journal of Neurology, 23(2), 387–392. 10.1111/ene.12878 26518336

[R13] Chu HY, McIver EL, Kovaleski RF, Atherton JF, & Bevan MD (2017). Loss of hyperdirect pathway cortico-subthalamic inputs following degeneration of midbrain dopamine neurons. Neuron, 95(6), 1306–1318 e1305. 10.1016/j.neuron.2017.08.038 28910619 PMC5679443

[R14] Connolly BS, & Lang AE (2014). Pharmacological treatment of Parkinson disease: A review. Jama, 311(16), 1670–1683. 10.1001/jama.2014.3654 24756517

[R15] Cross ZR., Chatburn A., Melberzs L., Temby P., Pomeroy D., Schlesewsky M., & Bornkessel-Schlesewsky I. (2022). Task-related, intrinsic oscillatory and aperiodic neural activity predict performance in naturalistic team-based training scenarios. Scientific Reports, 12(1), 16172. 10.1038/s41598-022-20704-8 36171478 PMC9519541

[R16] Darmani G, Drummond NM, Ramezanpour H, Saha U, Hoque T, Udupa K, Sarica C, Zeng K, Cortez Grippe T, Nankoo JF, Bergmann TO, Hodaie M, Kalia SK, Lozano AM, Hutchison WD, Fasano A, & Chen R. (2023). Long-term recording of subthalamic aperiodic activities and beta bursts in Parkinson’s disease. Movement Disorders, 38(2), 232–243. 10.1002/mds.29276 36424835

[R17] Delorme A, & Makeig S. (2004). EEGLAB: An open source toolbox for analysis of single-trial EEG dynamics including independent component analysis. Journal of Neuroscience Methods, 134(1), 9–21. 10.1016/j.jneumeth.2003.10.009 15102499

[R18] Donoghue T, Dominguez J,& Voytek B(2020). Electrophysiological frequency band ratio measures conflate periodic and aperiodic neural activity. eNeuro, 7(6), 1–14. 10.1523/ENEURO.0192-20.2020 PMC7768281 32978216

[R19] Donoghue T, Haller M, Peterson EJ, Varma P, Sebastian P, Gao R, Noto T, Lara AH, Wallis JD, Knight RT, Shestyuk A, & Voytek B. (2020). Parameterizing neural power spectra into periodic and aperiodic components. Nature Neuroscience, 23(12), 1655–1665. 10.1038/s41593-020-00744-x 33230329 PMC8106550

[R20] Finley AJ, Angus DJ, van Reekum CM, Davidson RJ, & Schaefer SM (2022). Periodic and aperiodic contributions to theta-beta ratios across adulthood. Psychophysiology, 59(11), e14113. 10.1111/psyp.14113 35751645 PMC9532351

[R21] Firbank MJ, Parikh J, Murphy N, Killen A, Allan CL, Collerton D, Blamire AM, & Taylor JP (2018). Reduced occipital GABA in Parkinson disease with visual hallucinations. Neurology, 91(7), e675–e685. 10.1212/WNL.0000000000006007 30021920 PMC6105043

[R22] Gao R, Peterson EJ, & Voytek B. (2017). Inferring synaptic excitation/inhibition balance from field potentials. NeuroImage, 158, 70–78. 10.1016/j.neuroimage.2017.06.078 28676297

[R23] George JS, Strunk J, Mak-McCully R, Houser M, Poizner H, & Aron AR (2013). Dopaminergic therapy in Parkinson’s disease decreases cortical beta band coherence in the resting state and increases cortical beta band power during executive control. Neuroimage: Clinical, 3, 261–270. 10.1016/j.nicl.2013.07.013 24273711 PMC3814961

[R24] Gerster M, Waterstraat G, Litvak V, Lehnertz K, Schnitzler A, Florin E, Curio G, & Nikulin V. (2022). Separating neural oscillations from aperiodic 1/f activity: Challenges and recommendations. Neuroinformatics, 20(4), 991–1012. 10.1007/s12021-022-09581-8 35389160 PMC9588478

[R25] Gomez C, Olde Dubbelink KT, Stam CJ, Abasolo D, Berendse HW, & Hornero R. (2011). Complexity analysis of resting-state MEG activity in early-stage Parkinson’s disease patients. Annals of Biomedical Engineering, 39(12), 2935–2944. 10.1007/s10439-011-0416-0 21969108

[R26] Hill AT, Clark GM, Bigelow FJ, Lum JAG, & Enticott PG (2022). Periodic and aperiodic neural activity displays age-dependent changes across early-to-middle childhood. Developmental Cognitive Neuroscience, 54, 101076. 10.1016/j.dcn.2022.101076 35085871 PMC8800045

[R27] Hohaia W, Saurels BW, Johnston A, Yarrow K, & Arnold DH (2022). Occipital alpha-band brain waves when the eyes are closed are shaped by ongoing visual processes. Scientific Reports, 12(1), 1194. 10.1038/s41598-022-05289-6 35075196 PMC8786963

[R28] Immink MA, Cross ZR, Chatburn A, Baumeister J, Schlesewsky M, & Bornkessel-Schlesewsky I. (2021). Resting-state aperiodic neural dynamics predict individual differences in visuomotor performance and learning. Human Movement Science, 78, 102829. 10.1016/j.humov.2021.102829 34139391

[R29] Jaramillo-Jimenez A, Suarez-Revelo JX, Ochoa-Gomez JF, Carmona Arroyave JA, Bocanegra Y, Lopera F, Buriticá O, Pineda-Salazar DA, Moreno Gómez L, Tobón Quintero CA, Borda MG, Bonanni L, Ffytche DH, Brønnick K, & Aarsland D. (2021). Resting-state EEG alpha/theta ratio related to neuropsychological test performance in Parkinson’s disease. Clinical Neurophysiology, 132(3), 756–764. 10.1016/j.clinph.2021.01.001 33571883

[R30] Jung TP, Makeig S, Humphries C, Lee TW, McKeown MJ, Iragui V, & Sejnowski TJ (2000). Removing electroencephalographic artifacts by blind source separation. Psychophysiology, 37(2), 163–178. 10.1111/1469-8986.3720163 10731767

[R31] Karalunas SL, Ostlund BD, Alperin BR, Figuracion M, Gustafsson HC, Deming EM, Foti D, Antovich D, Dude J, Nigg J, & Sullivan E. (2022). Electroencephalogram aperiodic power spectral slope can be reliably measured and predicts ADHD risk in early development. Developmental Psychobiology, 64(3), e22228. 10.1002/dev.22228 35312046 PMC9707315

[R32] Kim J, Lee J, Kim E, Choi JH, Rah JC, & Choi JW (2022). Dopamine depletion can be predicted by the aperiodic component of subthalamic local field potentials. Neurobiology of Disease, 168, 105692. 10.1016/j.nbd.2022.105692 35306174

[R33] Kuhn AA, Kupsch A, Schneider GH, & Brown P. (2006). Reduction in subthalamic 8–35 Hz oscillatory activity correlates with clinical improvement in Parkinson’s disease. European Journal of Neuroscience, 23(7), 1956–1960. 10.1111/j.1460-9568.2006.04717.x 16623853

[R34] Kuhn AA, Tsui A, Aziz T, Ray N, Brucke C, Kupsch A, Schneider GH, & Brown P. (2009). Pathological synchronisation in the subthalamic nucleus of patients with Parkinson’s disease relates to both bradykinesia and rigidity. Experimental Neurology, 215(2), 380–387. 10.1016/j.expneurol.2008.11.008 19070616

[R35] Lempel A, & Ziv J. (1976). On the complexity of finite sequences. IEEE Transactions on Information Theory, 22(1), 75–81.

[R36] Little S, & Brown P. (2014). The functional role of beta oscillations in Parkinson’s disease. Parkinsonism & Related Disorders, 20(Suppl. 1), S44–S48. 10.1016/S1353-8020(13)70013-0 24262186

[R37] Manning JR, Jacobs J, Fried I, & Kahana MJ (2009). Broadband shifts in local field potential power spectra are correlated with single-neuron spiking in humans. Journal of Neuroscience, 29(43), 13613–13620. 10.1523/JNEUROSCI.2041-09.2009 19864573 PMC3001247

[R38] Marras C, Beck JC, Bower JH, Roberts E, Ritz B, Ross GW, Ross GW, Abbott RD, Savica R, Van Den Eeden SK, Willis AW, Tanner CM, & Parkinson’s Foundation PG (2018). Prevalence of Parkinson’s disease across North America. NPJ Parkinsons Disease, 4, 21. 10.1038/s41531-018-0058-0 PMC6039505 30003140

[R39] Martin S., Iturrate I., Chavarriaga R., Leeb R., Sobolewski A., Li AM., Zaldivar J., Peciu-Florianu I., Pralong E., Castro-Jiménez M., Benninger D., Vingerhoets F., Knight RT., Bloch J., & Millan JDR. (2018). Differential contributions of subthalamic beta rhythms and 1/f broadband activity to motor symptoms in Parkinson’s disease. NPJ Parkinsons Disease, 4, 32. 10.1038/s41531-018-0068-y PMC6218479 30417084

[R40] Meder D, Herz DM, Rowe JB, Lehericy S, & Siebner HR (2019). The role of dopamine in the brain—Lessons learned from Parkinson’s disease. NeuroImage, 190, 79–93. 10.1016/j.neuroimage.2018.11.021 30465864

[R41] Merkin A, Sghirripa S, Graetz L, Smith AE, Hordacre B, Harris R, Pitcher J, Semmler J, Rogasch NC, & Goldsworthy M. (2023). Do age-related differences in aperiodic neural activity explain differences in resting EEG alpha? Neurobiology of Aging, 121, 78–87. 10.1016/j.neurobiolaging.2022.09.003 36379095

[R42] Miladinović A, Ajčević M, Busan P, Jarmolowska J, Deodato M, Mezzarobba S, Battaglini PP, & Accardo A. (2021). EEG changes and motor deficits in Parkinson’s disease patients: Correlation of motor scales and EEG power bands. Procedia Computer Science, 192, 2616–2623. 10.1016/j.procs.2021.09.031

[R43] Moustafa AA, Cohen MX, Sherman SJ, & Frank MJ (2008). A role for dopamine in temporal decision making and reward maximization in parkinsonism. Journal of Neuroscience, 28(47), 12294–12304. 10.1523/JNEUROSCI.3116-08.2008 19020023 PMC3049941

[R44] O’Gorman Tuura RL, Baumann CR, & Baumann-Vogel H. (2018). Beyond dopamine: GABA, glutamate, and the axial symptoms of Parkinson disease. Frontiers in Neurology, 9, 806. 10.3389/fneur.2018.00806 30319535 PMC6168661

[R45] Olde Dubbelink KT, Stoffers D, Deijen JB, Twisk JW, Stam CJ, & Berendse HW (2013). Cognitive decline in Parkinson’s disease is associated with slowing of resting-state brain activity: A longitudinal study. Neurobiology of Aging, 34(2), 408–418. 10.1016/j.neurobiolaging.2012.02.029 22495052

[R46] Ostlund BD, Alperin BR, Drew T, & Karalunas SL (2021). Behavioral and cognitive correlates of the aperiodic (1/f-like) exponent of the EEG power spectrum in adolescents with and without ADHD. Developmental Cognitive Neuroscience, 48, 100931. 10.1016/j.dcn.2021.100931 33535138 PMC7856425

[R47] Ouyang G, Hildebrandt A, Schmitz F, & Herrmann CS (2020). Decomposing alpha and 1/f brain activities reveals their differential associations with cognitive processing speed. NeuroImage, 205, 116304. 10.1016/j.neuroimage.2019.116304 31654760

[R48] Remple MS, Bradenham CH, Kao CC, Charles PD, Neimat JS, & Konrad PE (2011). Subthalamic nucleus neuronal firing rate increases with Parkinson’s disease progression. Movement Disorders, 26(9), 1657–1662. 10.1002/mds.23708 21542021 PMC3151310

[R49] Rosenblum Y, Shiner T, Bregman N, Giladi N, Maidan I, Fahoum F, & Mirelman A. (2023). Decreased aperiodic neural activity in Parkinson’s disease and dementia with Lewy bodies. Journal of Neurology, 270(8), 3958–3969. 10.1007/s00415-023-11728-9 37138179

[R50] Sanchez-Ferro A, Matarazzo M, Martinez-Martin P, Martinez-Avila JC, Gomez de la Camara A, Giancardo L, Gallego TA, Montero P, Puertas-Martín V, Obeso I, Butterworth I, Mendoza CS, Catalán MJ, Molina JA, Bermejo-Pareja F, Martínez-Castrillo JC, López-Manzanares L, Alonso-Cánovas A, Rodríguez JH, & Gray M. (2018). Minimal clinically important difference for UPDRS-III in daily practice. Movement Disorders Clinical Practice, 5(4), 448–450. 10.1002/mdc3.12632 30838303 PMC6336372

[R51] Schneider B, Szalardy O, Ujma PP, Simor P, Gombos F, Kovacs I, Dresler M, & Bodizs R. (2022). Scale-free and oscillatory spectral measures of sleep stages in humans. Frontiers in Neuroinformatics, 16, 989262. 10.3389/fninf.2022.989262 36262840 PMC9574340

[R52] Shimamoto SA, Ryapolova-Webb ES, Ostrem JL, Galifianakis NB, Miller KJ, & Starr PA (2013). Subthalamic nucleus neurons are synchronized to primary motor cortex local field potentials in Parkinson’s disease. Journal of Neuroscience, 33(17), 7220–7233. 10.1523/JNEUROSCI.4676-12.2013 23616531 PMC3673303

[R53] Shuffrey LC, Pini N, Potter M, Springer P, Lucchini M, Rayport Y, Sania A, Firestein M, Brink L, Isler JR, Odendaal H, & Fifer WP (2022). Aperiodic electrophysiological activity in preterm infants is linked to subsequent autism risk. Developmental Psychobiology, 64(4), e22271. 10.1002/dev.22271 35452546 PMC9169229

[R54] Singh A, Richardson SP, Narayanan N, & Cavanagh JF (2018). Mid-frontal theta activity is diminished during cognitive control in Parkinson’s disease. Neuropsychologia, 117, 113–122. 10.1016/j.neuropsychologia.2018.05.020 29802866 PMC6524769

[R55] Song Y, Gong T, Xiang Y, Mikkelsen M, Wang G, & Edden RAE (2021). Single-dose L-dopa increases upper brainstem GABA in Parkinson’s disease: A preliminary study. Journal of the Neurological Sciences, 422, 117309. 10.1016/j.jns.2021.117309 33548666

[R56] Stoffers D, Bosboom JL, Deijen JB, Wolters EC, Berendse HW, & Stam CJ (2007). Slowing of oscillatory brain activity is a stable characteristic of Parkinson’s disease without dementia. Brain, 130(Pt 7), 1847–1860. 10.1093/brain/awm034 17412733

[R57] van Nuland AJM, den Ouden HEM, Zach H, Dirkx MFM, van Asten JJA, Scheenen TWJ, Toni I, Cools R, & Helmich RC (2020). GABAergic changes in the thalamocortical circuit in Parkinson’s disease. Human Brain Mapping, 41(4), 1017–1029. 10.1002/hbm.24857 31721369 PMC7267977

[R58] Vinding MC, Tsitsi P, Waldthaler J, Oostenveld R, Ingvar M, Svenningsson P, & Lundqvist D. (2020). Reduction of spontaneous cortical beta bursts in Parkinson’s disease is linked to symptom severity. Brain Communications, 2(1), fcaa052. 10.1093/braincomms/fcaa052 PMC7425382 32954303

[R59] Voytek B, Kramer MA, Case J, Lepage KQ, Tempesta ZR, Knight RT, & Gazzaley A. (2015). Age-related changes in 1/f neural electrophysiological noise. Journal of Neuroscience, 35(38), 13257–13265. 10.1523/JNEUROSCI.2332-14.2015 26400953 PMC4579381

[R60] Wang Z, Mo Y, Sun Y, Hu K, Peng C, Zhang S, & Xue S. (2022). Separating the aperiodic and periodic components of neural activity in Parkinson’s disease. European Journal of Neuroscience, 56(6), 4889–4900. 10.1111/ejn.15774 35848719

[R61] Waschke L, Donoghue T, Fiedler L, Smith S, Garrett DD, Voytek B, & Obleser J. (2021). Modality-specific tracking of attention and sensory statistics in the human electrophysiological spectral exponent. eLife, 10, 1–25. 10.7554/eLife.70068 PMC8585481 34672259

[R62] Weinberger M., Mahant N., Hutchison WD., Lozano AM., Moro E., Hodaie M., Lang AE., & Dostrovsky JO. (2006). Beta oscillatory activity in the subthalamic nucleus and its relation to dopaminergic response in Parkinson’s disease. Journal of Neurophysiology, 96(6), 3248–3256. 10.1152/jn.00697.2006 17005611

[R63] Winawer J, Kay KN, Foster BL, Rauschecker AM, Parvizi J, & Wandell BA (2013). Asynchronous broadband signals are the principal source of the BOLD response in human visual cortex. Current Biology, 23(13), 1145–1153. 10.1016/j.cub.2013.05.001 23770184 PMC3710543

[R64] Winkler I, Haufe S, & Tangermann M. (2011). Automatic classi- fication of artifactual ICA-components for artifact removal in EEG signals. Behavioral Brain Functions, 7, 30. 10.1186/1744-9081-7-30 21810266 PMC3175453

[R65] Yilmaz NH, Calisoglu P, Guntekin B, & Hanoglu L. (2020). Correlation between alpha activity and neuropsychometric tests in Parkinson’s disease. Neuroscience Letters, 738, 135346. 10.1016/j.neulet.2020.135346 32911456

