# Supplementary material for: Medication-invariant resting aperiodic and periodic neural activity in Parkinson’s disease
Source: Psychophysiology. Author manuscript; Available in PMC 2024 Nov 7. (PMC11542173; doi:10.1111/psyp.14478)
Supplement: Supplementary Materials — Figure S1. Correlation matrix for PD patients during EC condition. Reported are the r values. Figure S2. Correlation matrix for PD patients during EO condition. Reported are the r values. Figure S3. Correlation matrix for CTL during EC condition (top panel) and EO condition (bottom panel). Reported are the r values. Table S1. Medication status of Parkinson’s disease participants. [file NIHMS2027043-supplement-Supplementary_Materials.docx]

**Supplementary material #1**

***Correlations between clinical outcomes and EEG parameters***

Exploratory correlation analyses were performed to assess the association between each clinical outcome and EEG measure for PD patients during EC (Figure 1) and EO conditions (Figure 2), and for CTL during EC and EO conditions (Figure 3). Overall, those individuals with greater NAART scores also had greater levels of alpha and beta activity when adjusted for aperiodic activity. Furthermore, those individuals who had a larger difference in their medicated and not medicated motor impairment (UPDRS DIFF) also had smaller ON alpha power, however this was mitigated when accounting for aperiodic activity.

| **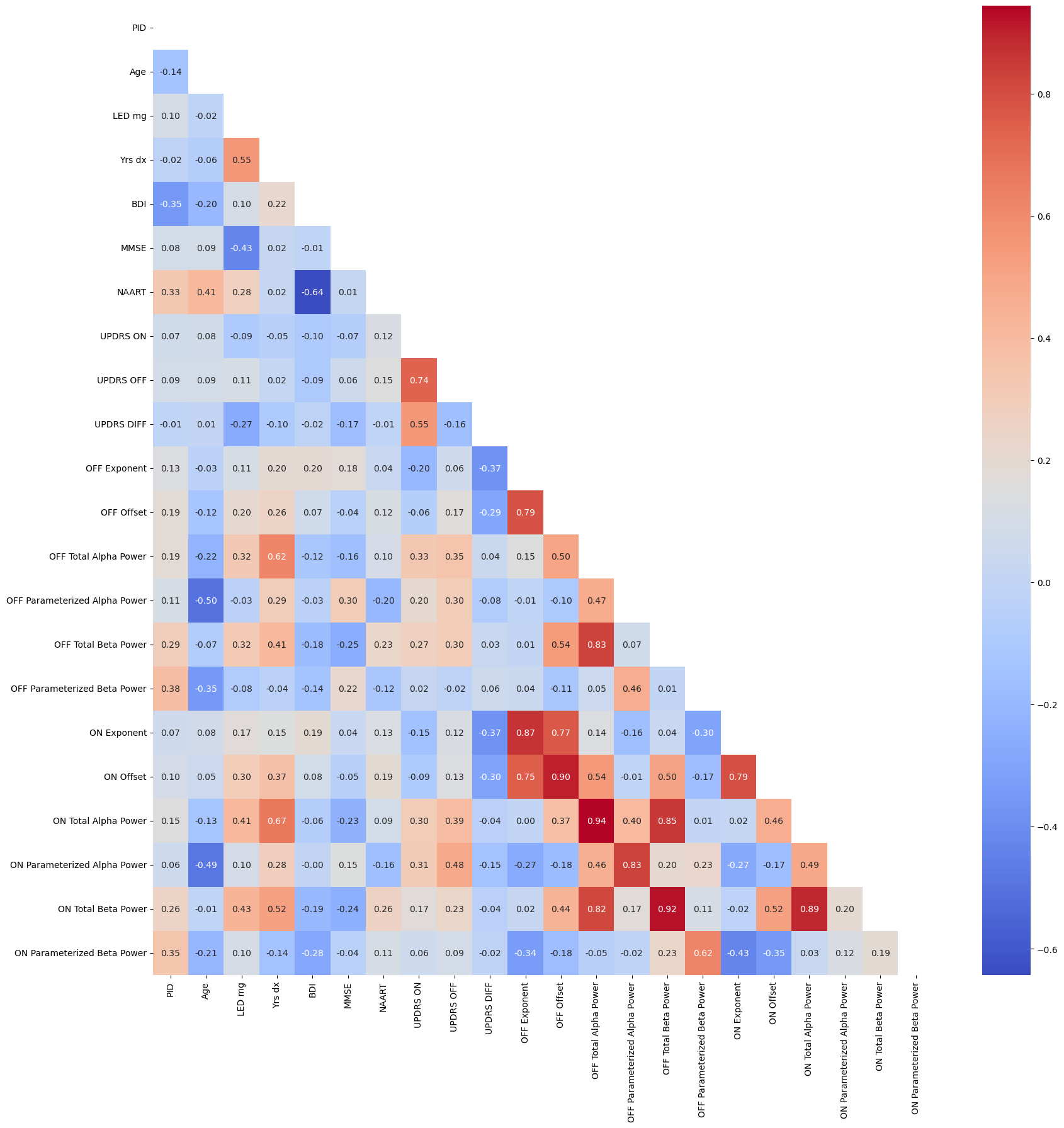** |
| --- |
| **Figure 1.** Correlation matrix for PD patients during EC condition. Reported are the r values. |

| **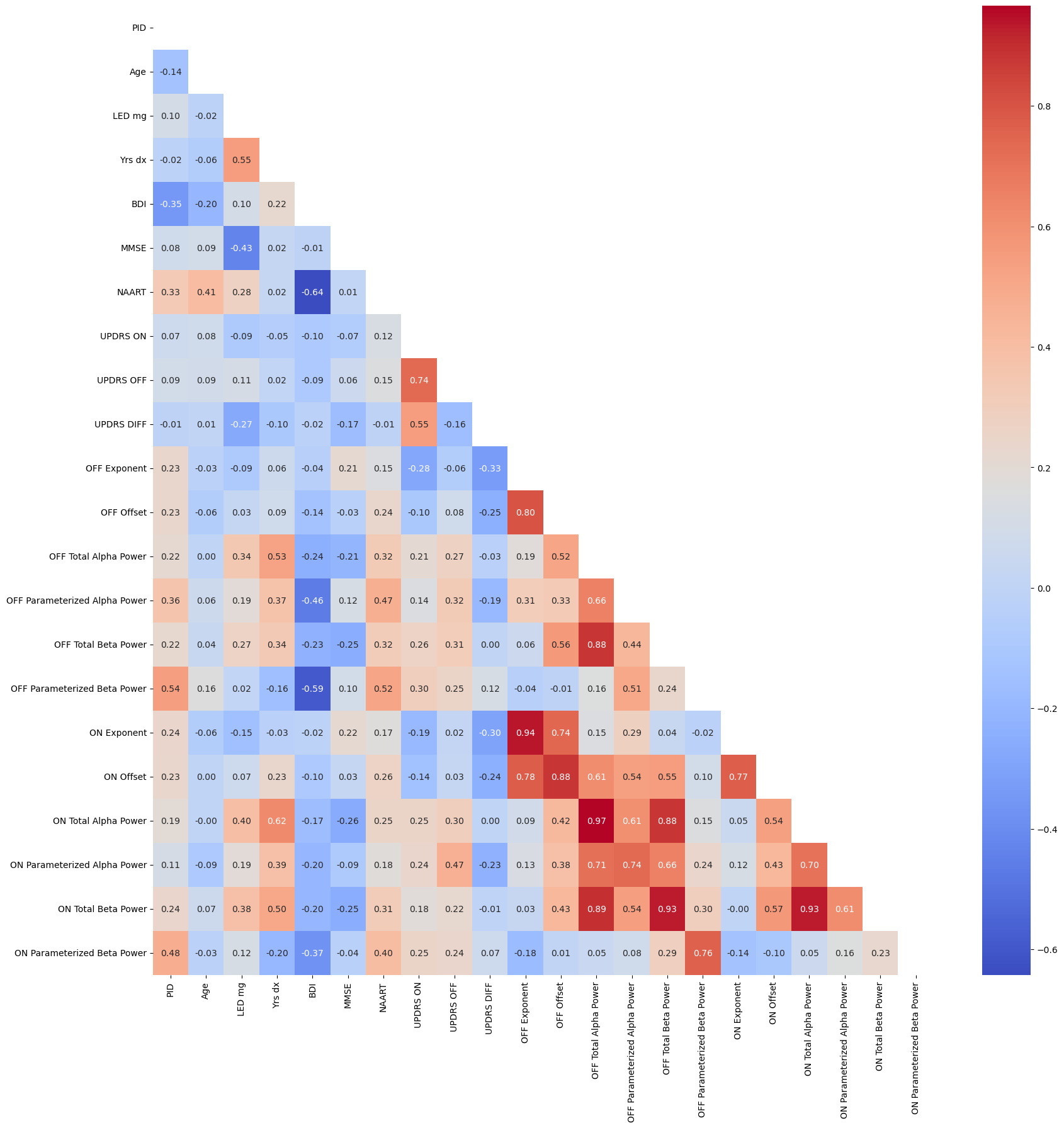** |
| --- |
| **Figure 2.** Correlation matrix for PD patients during EO condition. Reported are the r values. |

| **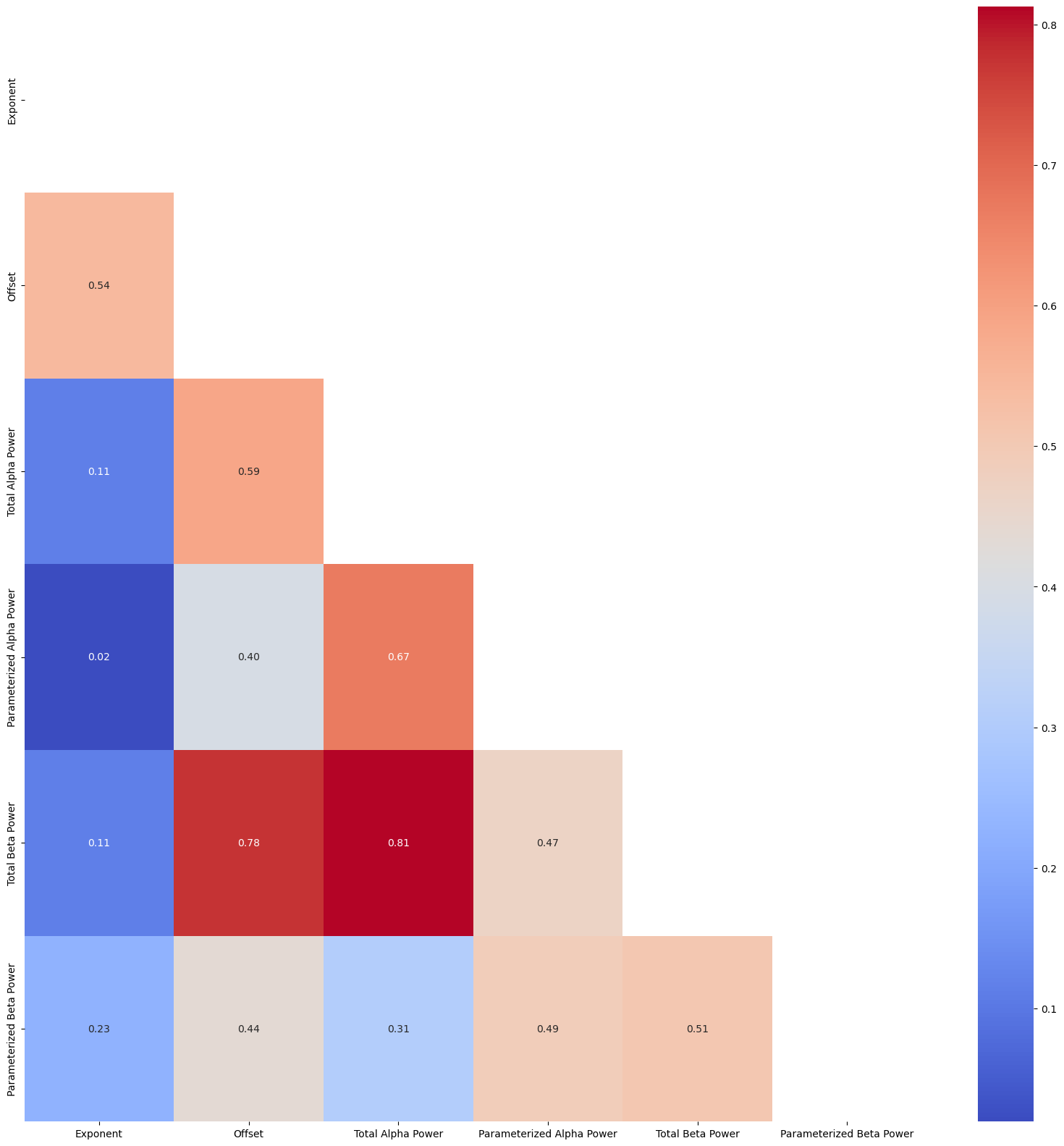** |
| --- |
| **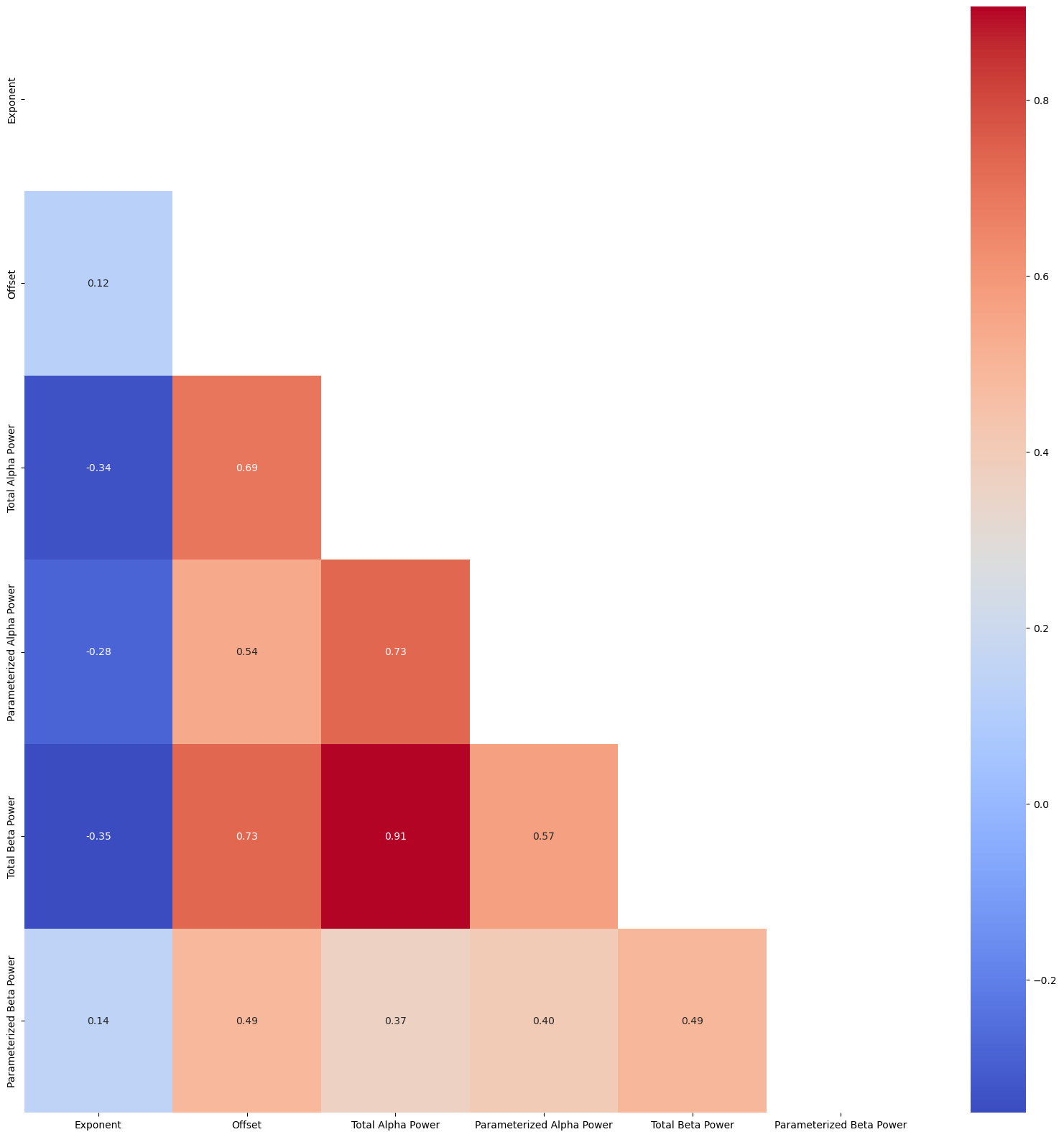** |
| **Figure 3.** Correlation matrix for CTL during EC condition (top panel) and EO condition (bottom panel). Reported are the r values. |

**Supplementary material #2**

*Table 1. Medication status of Parkinson’s Disease participants.*

| Class | Medication active ingredient | Dosage (mg) | Frequency (per day) | Subject no. |
| --- | --- | --- | --- | --- |
| Levodopa-PDDI |  |  |  |  |
|  | Levodopa-carbidopa | 10/100 – 25/100 | 1 – 7 | 26 |
| Dopamine agonists |  |  |  |  |
|  | Pramipexole | .125 – 1.5 | 1 – 3 | 4 |
|  | Ropinirole | 1 – 10 | 1 – 5 | 5 |
|  | Rotigotine | 8 | 1 | 1 |
| MAOBIs |  |  |  |  |
|  | Rasagiline | 1 – 10 | 1 | 4 |
|  | Selegiline | 5 – 10 | 1 – 4 | 4 |
| COMTIs |  |  |  |  |
|  | Amantadine | 100 | 1 | 1 |

*PDDI, peripheral dopa decarboxylase inhibitors; MAOBIs, monoamine oxidase type B inhibitors; COMTIs, catechol-O-methyltransferase inhibitors.*
